# Comparisons between comorbid conditions and health care consumption in rheumatoid arthritis patients with or without biological disease-modifying anti-rheumatic drugs: a register-based study

**DOI:** 10.1186/s12891-016-1354-7

**Published:** 2016-12-12

**Authors:** Karin Bengtsson, Lennart T. H. Jacobsson, Barbro Rydberg, Göran Kvist, Tomas Torstenson, Mats Dehlin, Elisabet Hilme, Anna Lindhé, Susanna Maria Wallerstedt, Helena Forsblad-d’Elia

**Affiliations:** 1 Department of Rheumatology and Inflammation Research, Sahlgrenska Academy at University of Gothenburg, Box 480, S-405 30, Gothenburg, Sweden; 2 Department of Rheumatology, Skövde Hospital (Kärnsjukhuset), Skövde, Sweden; 3 Department of Rheumatology, Södra Älvsborgs Hospital, Borås, Sweden; 4 Department of Rheumatology, Uddevalla Hospital, Uddevalla, Sweden; 5 Department of Rheumatology, Alingsås Hospital, Alingsås, Sweden; 6 Regional Health Care, Västra Götaland, Gothenburg, Sweden; 7 Department of Clinical Pharmacology, Sahlgrenska University Hospital and Sahlgrenska Academy, University of Gothenburg, Gothenburg, Sweden; 8 Departments of Public Health and Clinical Medicine, Rheumatology, Umeå University, Umeå, Sweden

**Keywords:** Rheumatoid arthritis, Comorbidity, Health care consumption, Biological therapy, bDMARDs, DMARDs, Biologic agents

## Abstract

**Background:**

Symptoms and prognosis of patients with rheumatoid arthritis (RA) have improved with more intensive therapy, including the biological disease-modifying anti-rheumatic drugs (bDMARDs). Real life data concerning how comorbidities are distributed among patients treated or not treated with bDMARDs are scarce. Our objective was to investigate differences in comorbidity and health care consumption in RA patients, with and without bDMARDs.

**Methods:**

This cross-sectional study was performed in the Southwestern part of Sweden. Patients, aged ≥ 18 years and diagnosed with RA in secondary health care during 2009–2010, were identified in the regional health care database. Aggregated data of comorbidity and health care consumption were retrieved between 2006 and 2010. RA patients treated with bDMARDs on 31st December 2010 were identified in the Swedish Rheumatology Quality Register (SRQ), which includes the biologics register Anti-Rheumatic Therapy in Sweden (ARTIS). Descriptive, comparative, univariate and multiple logistic regression analyses were used to identify factors associated with bDMARDs.

**Results:**

Seven thousand seven hundred and twelve (7712) RA patients were identified (age 64.8 ± 14.9 years, women 74.3%), of whom 1137 (14.7%) were treated with bDMARDs. Overall, the most common comorbidities were infections (69.2%), hypertension (41.1%), chronic respiratory disease (15.3%), ischemic heart disease (14.0%) and malignancy (13.7%). Patients without bDMARDs were older and had more comorbidity. In the multiple logistic regression analysis, older age, cerebrovascular and chronic respiratory disease, heart failure, depression and malignancy were all associated with no present bDMARDs. Infections were associated with bDMARDs. Patients treated with bDMARDs consumed more secondary outpatient care but less visits in primary health care compared to patients without bDMARDs.

**Conclusions:**

Patients treated with bDMARDs versus no bDMARDs were younger and had significantly lower period prevalence for most common comorbidities, with the exception of infections. Differences in comorbidities between RA patients with or without bDMARDs should be taken into consideration when evaluating effectiveness and safety of bDMARDs in ordinary care.

**Electronic supplementary material:**

The online version of this article (doi:10.1186/s12891-016-1354-7) contains supplementary material, which is available to authorized users.

## Background

Rheumatoid arthritis (RA) is a chronic inflammatory disease, affects more women than men and has a peak age of onset at the fifth to sixth decade of life [1]. Symptoms and prognosis of RA patients have dramatically improved over the last decades with more intensive therapy, including the introduction of the TNF inhibitors and other biological disease-modifying anti-rheumatic drugs (bDMARDs), such as anakinra (interleukin-1 receptor inhibitor), rituximab (monoclonal antibody against CD20 on B-cells), abatacept (targeting T-cells activation) and tocilizumab (interleukin-6 receptor inhibitor) [2, 3]. The use of bDMARDs is steadily increasing and bDMARDs are used in more early phases of the disease [4].

It is well known that RA is associated with both higher morbidity and mortality [5–10]. Several studies have shown an increased risk of cardiovascular disease (CVD) [11–15], which cannot fully be explained by traditional risk factors [16, 17]. Systemic inflammation is considered to contribute to the increased risk through accelerating the atherosclerotic process [18–20]. Studies have indicated that treatment reducing the systemic inflammation, such as TNF inhibitors, may reduce the cardiovascular risk [5, 21, 22]. However, observational studies like these are all likely to have some residual confounding by indication despite different attempts to overcome this. Such bias is, for example, likely for comorbidity that constitutes an absolute or relative contraindication for initiating bDMARDs therapy, i.e. heart failure, malignant disease and severe infections. There may also be other comorbidities and factors that clinicians may take into account before initiating therapy with bDMARDs. In addition, bDMARDs may themselves result in an increased risk of opportunistic as well as other infections.

In the light of this background, the primary aim of this study was to investigate the use of bDMARDs in patients with RA in the Southwestern part of Sweden and to see whether demographics (age, sex), comorbidities and health care consumption differed between patients treated with or without bDMARDs.

## Methods

### Setting

This is a cross-sectional population-based analysis investigating RA patients treated with or without bDMARDs on 31st December 2010. We have used the Swedish biologics register Anti-Rheumatic Therapy in Sweden (ARTIS) and five years of aggregated data from the regional health care database, Vega. The study was performed in the Region Västra Götaland, which is located in the Southwest of Sweden. On 31st December 2010, the region had 1,259,004 residents ≥ 18 years of age, representing 16.8% of the total Swedish population ≥ 18 years of age [23]. There are five hospitals with rheumatology clinics in the region, including Sahlgrenska University Hospital in Gothenburg. There is universal access to publicly funded health care for all Swedish residents. Practically all RA patients in Sweden are diagnosed and treated at a specialist rheumatology clinic. According to a previous study, the RA patients have their first appointment within a median time of three to four weeks after referral to the rheumatology clinic [24]. RA patients treated with bDMARDs are registered in the Swedish Rheumatology Quality register (SRQ), which contains the ARTIS. The national coverage of RA patients treated with bDMARDs in the ARTIS has in a recent report been estimated to 95% for the period 2008–2010 [25].

The regional health care data base, Vega, is an administrative health care register in the Region Västra Götaland and contains diagnoses registered within medical records in this region as well as demographics (sex, age, place of residence). Vega captures data from inpatient and outpatient care, both primary and secondary care, within public health care as well as private settings. Details are given concerning care providers, primary and secondary diagnoses according to International Classification of Diseases version 10 (ICD-10), and dates of physician visit, admission and discharge. According to a Swedish classification, held by the National Board of Health and Welfare, the data base also includes surgical and therapeutic procedure codes.

### Patients

#### Patients treated with bDMARDs

We identified all patients with RA or inflammatory polyarthritis, aged ≥ 18 years and present treatment with bDMARDs on 31st December 2010 according to data from ARTIS. With regard to the rheumatic disease and treatment with bDMARDs, the data from ARTIS were validated systematically against the medical records. Patients without RA diagnosis and/or without present treatment with bDMARDs in the medical records were excluded. Treatment with rituximab was considered as present if the therapy was given less than two years before 31st December 2010. Disease- and treatment-specific data was extracted from ARTIS; disease duration, presence of anti-citrullinated protein antibody (ACPA) and rheumatoid factor (RF), erosive disease, details of present bDMARDs, the number of previous bDMARDs, concomitant conventional synthetic disease-modifying anti-rheumatic drugs (csDMARDs), glucocorticosteroids, erythrocyte sedimentation rate (ESR), C-reactive protein (CRP), Disease Activity Score of 28 joints based on ESR and CRP (DAS28 and DAS28CRP respectively) [26] and Health Assessment Questionnaire disability index (HAQ-DI) [27]. The extracted data, such as ACPA, RF, erosive disease and treatment-specific data, were quality-controlled against medical records. The information was corrected when indicated. No imputations of remaining missing data were performed.

The patients identified in ARTIS were linked to Vega using the unique personal identification number that all Swedish residents have.

#### Patients without bDMARDs

To assemble a contemporary RA population without present bDMARDs on 31st December 2010, we used health care data from Vega. First, we identified all patients aged ≥ 18 years registered with a RA diagnosis in any secondary health care at least once between 1st January 2009 and 31st December 2010. ICD-10 codes M05 and M06, except M061 (adult-onset Still’s disease) and M064 (inflammatory polyarthritis), were used to define the RA diagnosis. Second, the RA patients found in Vega and not simultaneously identified in the ARTIS extraction as RA patients with present bDMARDs were classified as “without bDMARDs”. No disease- and treatment-specific data was available for RA patients without bDMARDs.

For all identified RA patients (with and without bDMARDs), health care data from January 2006 until December 2010 were extracted. Patients residing outside the Region Västra Götaland during the entire extraction period in Vega were excluded.

### Comorbidity and health care consumption

Comorbid conditions and health care consumption were retrieved for the period 2006 to 2010. To capture both common and less frequent comorbid conditions this five-year period of historical register data was chosen. The comorbid conditions identified were diabetes mellitus, hypertension, ischemic heart disease, heart failure, valvular disease, atrial fibrillation or flutter, cerebrovascular disease, venous thromboembolic disease, chronic respiratory disease, chronic renal insufficiency, depression, malignancy, infections, fractures at sites related to osteoporosis and prosthetic surgery. Comorbid conditions were based on ICD-10 codes registered at physician visits in primary and secondary health care or as discharge diagnoses after hospitalization. The used ICD-10 codes are described in detail in the Additional file 1: Table S1. The percentage of patients hospitalized at least once, as well as the number of admissions and cumulative days of inpatient care at hospitals were analysed. Hospitalization of one day with a registered therapeutic procedure code of intravenous therapy could be solely due to the administration of intravenous bDMARDs. These hospitalizations were therefore excluded. The number of physician visits in primary and secondary outpatient care were computed and presented separately.

### Statistics

Descriptive statistics are presented as number (percentage), mean ± standard deviation (SD) or median (first, third quartile). Comparisons between groups were assessed by T-test or Mann-Whitney U test, chi-square test or, when applicable, Fischer’s exact test. Univariate and multiple logistic regression analyses with bDMARDs (1) or not (0) as the dependent variable were used to identify factors associated with usage of bDMARDs. Comorbid covariates were included in the models if they differed significantly between the two groups in the comparative analyses. In the univariate logistic regression analyses, each covariate was also adjusted for sex and age. All tests were two-tailed and *p* < 0.05 was considered statistically significant. Statistical analyses were performed by PASW Statistics 19 (SPSS Inc., IBM, Chicago, USA) and SAS Version 9.3 (SAS Institute Inc., Cary, North Carolina, USA)

## Results

**Fig. 1:**
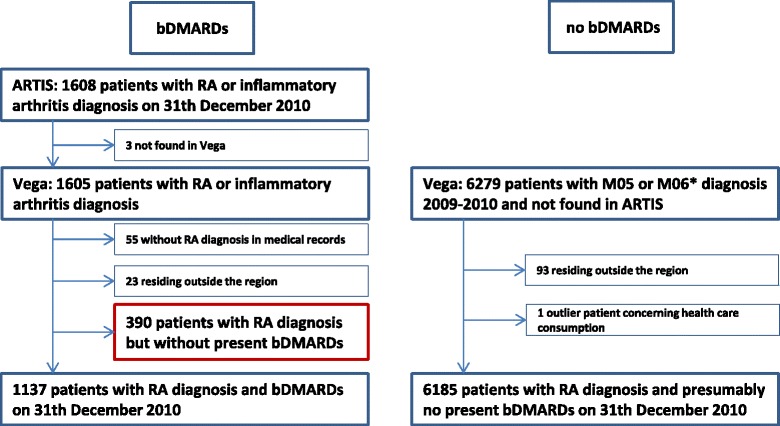
Flow chart of the inclusion and exclusion process. ARTIS = Anti-Rheumatic Therapy in Sweden; the Swedish biologic register. Vega = the regional health care data base. RA = rheumatoid arthritis. bDMARDs = biologic disease-modifying anti-rheumatic drugs. * M061 and M064 not included

In ARTIS, 1608 patients were first identified with present bDMARDs, and 1137 of these patients met the inclusion criteria after validation (Fig. 1). Three-hundred and ninety patients had a RA diagnosis but no present treatment with bDMARDs; 311 of these patients had a history of previous bDMARDs and 79 patients were planned to begin with a bDMARDs in 2011. These 390 RA patients were not included in the comparative analyses. In Vega, initially 6279 patients were identified without bDMARDs, and 6185 of these met the inclusion criteria (Fig. 1).

The characteristics of the RA patients treated with bDMARDs are presented in Table 1. The mean age of the patients was 58.9 years and 77.4% of them were females. The patients had a longstanding, often RF positive and erosive, disease. TNF inhibitors represented approximately 3/4 of the bDMARDs. DAS28 and HAQ were high before initiating the bDMARDs and had decreased at follow-up.

**Table 1:** Demographics and disease-characteristics of the 1137 RA patients treated with bDMARDs

Demographics and disease-characteristics	
Women	880 (77.4)
Age, years	58.9 ± 12.3
Disease duration, years	16.2 ± 11.0
RF positive (*n* = 1132)	981 (86.7)
ACPA positive (*n* = 360)	297 (82.5)
Erosive disease (*n* = 1130)	980 (86.7)
DAS28, before start on present bDMARDs (*n* = 808)	5.16 ± 1.23
DAS28, last follow-up on present bDMARDs (*n* = 814)	3.02 ± 1.26
HAQ score, before start on present bDMARDs (*n* = 854)	1.29 ± 0.64
HAQ score, last follow-up on present bDMARDs (*n* = 839)	0.93 ± 0.65
Present bDMARDs	
TNF-inhibitor (infliximab, etanercept, adalimumab, certolizumab, golimumab)	881 (77.5)
Rituximab	183 (16.1)
Tocilizumab	56 (4.9)
Abatacept	13 (1.1)
Anakinra	4 (0.4)
Concomitant csDMARDs and glucocorticosteroids at last follow-up (*n* = 983)	
Any csDMARDs	826 (84.0)
Methotrexate	749 (76.2)
Glucocorticosteroids (prednisolone)	284 (28.9)
Numerical number of bDMARDs	
1	683 (60.1)
2	319 (28.1)
3	97 (8.5)
≥4	38 (3.3)

Data are expressed as mean ± SD or number (%). Data are presented for 1137 patients, but when data are missing, the numbers of patients are given. RF = rheumatoid factor, ACPA = anti-citrullinated protein antibody, DAS28 = disease activity score of 28 joints, HAQ = health assessment questionnaire, bDMARDs = biological disease-modifying anti-rheumatic drugs, csDMARDs = conventional synthetic disease-modifying anti-rheumatic drugs

### Description and comparison of comorbid conditions and health care consumption in RA patients with or without bDMARDs

In Table 2, descriptive and comparative analyses of comorbid conditions and health care consumption during the five-year period are displayed. A larger proportion of males and significantly older age were noted for patients without bDMARDs. The period prevalence of most of the comorbid conditions was significantly higher in patients without bDMARDs than in patients treated with bDMARDs. On the contrary, infections were more common in patients treated with bDMARDS than in patients without bDMARDs. The proportion of patients being hospitalized as well as the number of admissions did not differ significantly between those treated and not treated with bDMARDs. Longer hospitalizations were noted for patients without bDMARDs compared with patients treated with bDMARDs. Outpatient physician visits in secondary care were more frequent in patients treated with bDMARDs in comparison with patients without bDMARDs, while the opposite was noted for primary health care visits.

**Table 2:** Comorbidity and health care consumption 2006–2010 in RA patients with and without bDMARDs

	All patients (*n* = 7712)	Patients on bDMARDs (*n* = 1137)	Patients without bDMARDs (*n* = 6185)	*P*-value (bio vs no bio)
Women, n (%)	5727 (74.3)	880 (77.4)	4559 (73.7)	0.009
Age, years	64.8 ± 14.9	58.9 ± 12.3	66.2 ± 15.1	<0.001
Comorbid conditions, *n* (%)				
Diabetes mellitus	860 (11.2)	93 (8.2)	715 (11.6)	0.001
Hypertension	3169 (41.1)	359 (31.6)	2662 (43.0)	<0.001
Ischemic heart disease	1077 (14.0)	92 (8.1)	947 (15.3)	<0.001
Unstable angina Myocardial infarction Chronic ischemic heart disease	145 (1.9) 345 (4.5) 809 (10.5)	14 (1.2) 22 (1.9) 67 (5.9)	124 (2.0) 307 (5.0) 711 (11.5)	0.078 <0.001 <0.001
Heart failure	715 (9.3)	33 (2.9)	656 (10.6)	<0.001
Valvular disease	250 (3.2)	23 (2.0)	218 (3.5)	0.009
Atrial fibrillation or flutter	722 (9.4)	50 (4.4)	645 (10.4)	<0.001
Cerebrovascular disease	653 (8.5)	45 (4.0)	574 (9.3)	<0.001
Ischemic stroke Hemorrhagic stroke Unspecified stroke TIA	266 (3.4) 51 (0.7) 144 (1.9) 180 (2.3)	15 (1.3) 5 (0.4) 4 (0.4) 14 (1.2)	235 (3.8) 43 (0.7) 132 (2.1) 155 (2.5)	<0.001 0.327 <0.001 0.009
Venous thromboembolic disease Pulmonary embolus Deep venous thrombosis	424 (5.5) 116 (1.5) 141 (1.8)	47 (4.1) 5 (0.4) 19 (1.7)	363 (5.9) 109 (1.8) 118 (1.9)	0.019 0.001 0.588
Chronic respiratory disease	1178 (15.3)	130 (11.4)	984 (15.9)	<0.001
COPD and chronic bronchitis COPD and asthma Interstitial lung disease	627 (8.1) 1010 (13.1) 68 (0.9)	64 (5.6) 104 (9.1) 12 (1.1)	528 (8.5) 855 (13.8) 49 (0.8)	0.001 <0.001 0.370
Chronic renal insufficiency	162 (2.1)	8 (0.7)	143 (2.3)	<0.001
Depression	888 (11.5)	104 (9.1)	738 (11.9)	0.007
Malignancy	1059 (13.7)	87 (7.7)	921 (14.9)	<0.001
Infections	5339 (69.2)	835 (73.4)	4213 (68.1)	<0.001
Pneumonia Sepsis	1168 (15.1) 221 (2.9)	145 (12.8) 20 (1.8)	954 (15.4) 187 (3.0)	0.020 0.018
Fractures (sites related to osteoporosis)	566 (7.3)	65 (5.7)	476 (7.7)	0.019
Prosthetic surgery				
Hip Knee	318 (4.1) 271 (3.5)	43 (3.8) 38 (3.3)	253 (4.1) 213 (3.4)	0.627 0.862
Health care consumption				
Patients hospitalized^a^, n (%)	4001 (51.9)	571 (50.2)	3201 (51.8)	0.341
Number of inpatient admissions^a^ Cumulative days at hospital^a^	2 (1, 3) 8 (3, 24)	2 (1, 3) 6 (3, 14)	2 (1, 3) 9 (3, 26)	0.637 <0.001
Outpatient visits, secondary care	13 (7, 21)	21 (14, 34)	11 (7, 18)	<0.001
Outpatient visits, primary care	8 (4, 14)	7 (3, 11)	9 (5, 15)	<0.001

Data are expressed as number (%), mean ± SD or median (first, third quartile). Numbers of admissions, cumulative days at hospital and outpatient visits are calculated on patients who have been hospitalized or have been in outpatient care, respectively, and not on all patients. TIA = transient ischemic attack, COPD = chronic obstructive pulmonary disease

^a^Hospitalizations of one day and connected to an intravenous treatment were not included

Women and men were also analysed separately in order to identify any sex-specific differences in comorbidity and health care consumption within each sex (Table 3). The pattern was similar, with older age and more comorbid conditions in patients without bDMARDs in both the male and female comparisons. Notably, in general men had a higher period prevalence of comorbid conditions than women, with the exception of depression, infections and fractures.

**Table 3:** Comorbidity and health care consumption 2006–2010, divided by sex and bDMARDs

	Women, bDMARDs (*n* = 880)	Women, no bDMARDs (*n* = 4559)	*P*-value	Men, bDMARDs (*n* = 257)	Men, no bDMARDs (*n* = 1626)	*P*-value
Age, years	58.7 ± 12.4	65.6 ± 15.6	<0.001	59.7 ± 12.0	67.6 ± 13.5	<0.001
Comorbid conditions, *n* (%)						
Diabetes mellitus	62 (7.0)	478 (10.5)	0.002	31 (12.1)	237 (14.6)	0.284
Hypertension	268 (30.5)	1932 (42.4)	<0.001	91 (35.4)	730 (44.9)	0.004
Ischemic heart disease	48 (5.5)	591 (13.0)	<0.001	44 (17.1)	356 (21.9)	0.082
Heart failure	21 (2.4)	416 (9.1)	<0.001	12 (4.7)	240 (14.8)	<0.001
Valvular disease	15 (1.7)	151 (3.3)	0.011	8 (3.1)	67 (4.1)	0.443
Atrial fibrillation or flutter	34 (3.9)	396 (8.7)	<0.001	16 (6.2)	249 (15.3)	<0.001
Cerebrovascular disease	30 (3.4)	392 (8.6)	<0.001	15 (5.8)	182 (11.2)	0.009
Venous thromboembolic disease	41 (4.7)	260 (5.7)	0.215	6 (2.3)	103 (6.3)	0.011
Chronic respiratory disease	91 (10.3)	702 (15.4)	<0.001	39 (15.2)	282 (17.3)	0.390
Chronic renal insufficiency	6 (0.7)	90 (2.0)	0.008	2 (0.8)	53 (3.3)	0.028
Depression	87 (9.9)	614 (13.5)	0.004	17 (6.6)	124 (7.6)	0.567
Malignancy	61 (6.9)	597 (13.1)	<0.001	26 (10.1)	324 (19.9)	<0.001
Infections	663 (75.3)	3153 (69.2)	<0.001	172 (66.9)	1060 (65.2)	0.587
Pneumonia Sepsis	107 (12.2) 15 (1.7)	676 (14.8) 114 (2.5)	0.039 0.155	38 (14.8) 5 (1.9)	278 (17.1) 73 (4.5)	0.357 0.057
Fractures (sites related to osteoporosis)	59 (6.7)	402 (8.8)	0.039	6 (2.3)	74 (4.6)	0.102
Prosthetic surgery						
Hip Knee	33 (3.8) 29 (3.3)	186 (4.1) 165 (3.6)	0.649 0.635	10 (3.9) 9 (3.5)	67 (4.1) 48 (3.0)	0.863 0.633
Health care consumption						
Patients hospitalized^a^, *n* (%)	449 (51.0)	2369 (52.0)	0.609	122 (47.5)	832 (51.2)	0.271
Number of inpatient admissions^a^ Cumulative days at hospital^a^	2 (1, 3) 6 (3, 13)	2 (1, 3) 9 (3, 26)	0.752 <0.001	2 (1, 3) 7 (3, 15)	2 (1, 3) 10 (4, 26)	0.784 0.013
Outpatient visits, secondary care	22 (15, 34)	11 (7, 18)	<0.001	20 (12, 32)	11 (7, 18)	<0.001
Outpatient visits, primary care	7 (3, 11)	9 (5, 15)	<0.001	6 (3, 11)	8 (4, 13)	<0.001

Data are expressed as number (%), mean ± SD or median (first, third quartile). Numbers of admissions, cumulative days at hospital or outpatient visits are calculated on patients who have been hospitalized or have been in outpatient care, respectively, and not on all patients

^a^Hospitalizations of one day and connected to an intravenous treatment were not included

In Table 4, the ten most common primary discharge diagnoses are listed. The RA diagnosis was the main reason for hospitalization in both RA patients with and without bDMARDs, and especially in the group treated with bDMARDs.

**Table 4:** The top ten primary discharge diagnoses in patients with and without bDMARDs 2006–2010

	Primary discharge diagnoses in RA patients with bDMARDs (*n* = 2205)	Primary discharge diagnoses in RA patients without bDMARDs (*n* = 13,206)
1	Rheumatoid arthritis 800 (36.3)	Rheumatoid arthritis 1483 (11.2)
2	Ischemic heart disease 56 (2.5)	Ischemic heart disease 626 (4.7)
3	Fractures 51 (2.3)	Fractures 585 (4.4)
4	Osteoarthritis 42 (1.9)	Pneumonia 469 (3.6)
5	Pneumonia 41 (1.9)	Chest pain 349 (2.6)
6	Chest pain 41 (1.9)	Atrial fibrillation 330 (2.5)
7	Atrial fibrillation 35 (1.6)	Heart failure 312 (2.4)
8	Childbirth 34 (1.5)	Osteoarthritis 297 (2.2)
9	Abdominal pain 31 (1.4)	Stroke (hemorrhagic or ischemic) 247 (1.9)
10	Cholecystitis and/or gall stone 22 (1.0)	Urinary tract infection 236 (1.8)
	*Stroke 21 (1.0)*	*Abdominal pain 231 (1.7)*
	*Urinary tract infection 17 (0.8)*	*Childbirth 172 (1.3)*
	*Heart failure 6 (0.3)*	*Cholecystitis and/or gall stone 121 (0.9)*

Data are expressed as number (%). *N* = number of hospitalizations 2006–2010 when hospitalizations of one day and connected to an intravenous treatment, were not included. ICD-10 codes from the Z section are not included. In cursive, the corresponding number (%) is given in the other group if not already existing in the top ten

### Comorbid conditions and health care consumption associated with bDMARDs or not

In Table 5, the results of the age- and sex-adjusted univariate and the multiple logistic regression analyses are shown. The univariate analyses showed that cerebrovascular and ischemic heart disease, heart failure, atrial fibrillation, chronic respiratory disease, chronic renal insufficiency, malignancy and depression all occurred more frequently in those not treated with bDMARDs than in those treated with bDMARDs, whereas the occurrence of infections were more frequent in those treated with bDMARDs. Older age, but not sex, was associated with no present bDMARDs after the adjustment. The significantly longer hospitalization in RA patients without bDMARDs found in the comparative analysis disappeared after the age- and sex-adjustment.

**Table 5:** Associations with bDMARDs (1) or no bDMARDs (0), univariate and multiple logistic regression analyses

Covariates	Univariate analyses (sex- and age-adjusted)			Multiple analysis		
	Odds ratio	95% confidence intervals	*P*-value	Odds ratio	95% confidence intervals	*P*-value
Age (per 1 year)	0.97	0.97–0.97	<0.001	0.98	0.97–0.98	<0.001
Sex	0.88	0.76–1.03	0.103	0.94	0.81–1.10	0.450
Comorbid conditions						
Diabetes	0.86	0.69–1.09	0.218	0.96	0.76–1.22	0.758
Hypertension	0.89	0.76–1.03	0.108	0.95	0.81–1.11	0.513
Ischemic heart disease	0.73	0.58–0.93	0.009	0.90	0.70–1.15	0.384
Atrial fibrillation or flutter	0.64	0.47–0.86	0.004	0.83	0.60–1.15	0.260
Heart failure	0.40	0.28–0.58	<0.001	0.47	0.32–0.69	<0.001
Valvular disease	0.82	0.53–1.27	0.375	1.11	0.70–1.75	0.657
Cerebrovascular disease	0.59	0.43–0.82	0.001	0.65	0.47–0.90	0.010
Venous thromboembolic disease	0.83	0.61–1.14	0.259	0.92	0.67–1.27	0.607
Chronic respiratory disease	0.80	0.66–0.98	0.031	0.82	0.67–0.998	0.048
Chronic renal insufficiency	0.43	0.21–0.89	0.022	0.53	0.25–1.09	0.084
Depression	0.72	0.58–0.90	0.004	0.74	0.59–0.92	0.008
Malignancy	0.66	0.52–0.84	0.001	0.66	0.52–0.84	0.001
Infections	1.39	1.21–1.61	<0.001	1.52	1.31–1.76	<0.001
Fractures	1.01	0.77–1.33	0.947	1.06	0.81–1.41	0.660
Health care consumption						
Patients hospitalized^a^	1.15	1.01–1.31	0.040			
Number of inpatient admissions^a^	1.03	1.00–1.07	0.042			
Cumulative days at hospital^a^	0.997	0.99–1.00	0.101			
Outpatient visits, secondary care	1.06	1.06–1.07	<0.001			
Outpatient visits, primary care	0.97	0.96–0.98	<0.001			

Analyses were performed with (multiple) logistic regression models and expressed as Odds ratios (with 95% confidence intervals and *p*-value). In the univariate analyses, each covariate was adjusted for sex and age. Age and sex were adjusted for each other. In the multivariable analysis, all of the comorbid conditions covariates in the table were included in the model

^a^Hospitalizations of one day and connected to an intravenous treatment were not included

In the multivariable model, cerebrovascular and chronic respiratory disease, heart failure, malignancy, depression and older age were associated with no present bDMARDs, while infections were associated with bDMARDs.

## Discussion

In this register-based study of RA, which covers 1/6 of the Swedish adult population, we found a significantly higher frequency of comorbid conditions in RA patients not presently treated with bDMARDs compared to RA patients treated with bDMARDs, even after adjusting for the older age in RA patients without bDMARDs. RA patients treated with bDMARDs consumed more secondary health care, while the opposite were noted for RA patients without bDMARDS in primary health care.

Our main objective was to investigate differences of comorbid conditions and health care consumption in RA patients with or without bDMARDs. The design of this study did not allow us to address the causes of such differences. Several factors may thus have influenced the results of this cross-sectional analysis with five years of aggregated historical register data. Cerebrovascular and chronic respiratory disease, heart failure, depression, malignancy and older age were all associated with no present bDMARDs in this study. Confounding by indication due to absolute or relative contraindications for initiating bDMARDs therapy will automatically lead to lower frequency of malignancies and heart failure in the RA group treated with bDMARDs. Also, the older age and higher period prevalence of other comorbid conditions may suggest an increased fragility, which may have lead the treating physicians to avoid bDMARDs in these patients. This is in accordance with some previous studies that have demonstrated a lower use of bDMARDs in the elderly and that RA patients with less comorbidity were more likely to be treated with bDMARDs [28–32]. Infections were associated with bDMARDs in our study. This is in concordance with previous studies that have demonstrated an elevated risk of infections in patients with bDMARDs [7, 33]. In addition, part of the explanation may be that patients with bDMARDs in a higher degree are advised to seek health care in case of infectious symptoms and that severe RA is a risk factor for infections [34].

Studies specifically investigating the occurrence of comorbidities in the whole RA population, and not only in subgroups, are relatively uncommon. The differences in selection of patients, sex- and age-distribution, and collection of comorbidity data make it difficult to compare studies. A recent study by Dougados et al. reported a great variability of comorbidities in RA between countries, where this variability may partly be associated with the methods used to capture the information [35]. This study included RA patients from 17 different countries. The patients were younger than in our study and 39% had been exposed to bDMARDs. Compared to our results, Dougados et al. found similar prevalence of myocardial infarction and hypertension, but lower prevalence of malignancies and chronic obstructive pulmonary disease [35]. Likewise, even if not directly comparable due to different study designs, our findings concerning prevalence levels of the comorbid conditions are also in the same range as in other studies where prevalence of comorbidities have been reported in RA patients [36–39].

In the comparative analysis, we found a higher proportion of female RA patients with bDMARDs. The same result has been shown in other studies [39, 40]. However, in the multiple logistic regression analysis, we did not find sex to be associated with bDMARDs. This was probably a result of the higher burden of comorbidity that was found in male patients.

The disease characteristics in RA patients treated with bDMARDs were as expected in relation to the present recommendations regarding the initiation of bDMARDs. Furthermore, the health care consumption, in particular in secondary care, was high in the RA population treated with bDMARDs, and hospitalization often linked to the RA diagnosis per se.

There are several strengths of this present study. First, the large number of RA patients, which comprised 1/6 of the total Swedish adult population. The register-based study design has also assured that certain categories of patients, for example older patients, were not excluded. Second, the construction of our regional health care data base, including both inpatient care as well as primary and secondary outpatient care, enabled us to catch those comorbid conditions that do not necessarily need hospitalization or secondary outpatient care, such as hypertension, diabetes, chronic respiratory disease and depression. Third, our national biologic register ARTIS has an almost complete coverage of RA patients treated with bDMARDs [25].

There are limitations of the present study that must also be acknowledged. First, we cannot exclude misclassifications of diagnoses in the register and this is also the case for the RA diagnosis in the patients without bDMARDs. Though, our data gives a calculated RA period prevalence of 0.61% in our region, which is in accordance with previously published data from Sweden [31, 40]. The ICD-codes in Vega have not been validated previously. The secondary health care (secondary outpatient care and inpatient care) also report to the Swedish National Patient Register. The inpatient part of this register has been validated and the positive predictive value was found to differ between diagnoses but was generally high (85–95%) [41]. Second, RA patients only followed in primary health care and RA patients with physician visits less than every second year are not identified in our investigation. Third, the cross-sectional study design makes it impossible to draw conclusions of causality between comorbidities and health care consumption with respect to treatment with bDMARDs or not. Other limitations are the lack of information about disease- and treatment-specific characteristics of RA patients without bDMARDs.

## Conclusions

In this cross-sectional analysis with five years of aggregated historical register data, we show a clear difference between RA patients with and without bDMARDs concerning age, comorbid pattern and health care consumption. RA patients treated with bDMARDs were younger, had less comorbid conditions, and consumed more secondary outpatient care but less primary outpatient care. These differences in characteristics between RA patients with or without bDMARDs have to be taken into consideration when evaluating the effectiveness and safety of bDMARDs in ordinary care.

## Additional file

Additional file 1: Table S1. ICD 10 codes used to define comorbid conditions. (DOCX 19 kb)

## Abbreviations

ACPA: Anti-citrullinated protein antibody
ARTIS: Anti-rheumatic therapy in Sweden
bDMARDs: Biological disease-modifying anti-rheumatic drugs
CRP: C-reactive protein
csDMARDs: Conventional synthetic disease-modifying anti-rheumatic drugs
CVD: Cardiovascular disease
DAS28/DAS28CRP: Disease Activity Score of 28 joints based on ESR/CRP
DMARDs: Disease-modifying anti-rheumatic drugs
ESR: Erythrocyte sedimentation rate
HAQ-DI: Health Assessment Questionnaire disability index
ICD-10: International Classification of Diseases version 10
RA: Rheumatoid arthritis
RF: Rheumatoid factor
SD: Standard deviation
SRQ: Swedish Rheumatology Quality register
