# Supplementary material for: Comparisons between comorbid conditions and health care consumption in rheumatoid arthritis patients with or without biological disease-modifying anti-rheumatic drugs: a register-based study
Source: BMC Musculoskelet Disord. 2016 Dec 12;17:499. doi: 10.1186/s12891-016-1354-7 (PMC5154076; doi:10.1186/s12891-016-1354-7)
Supplement: Additional file 1: Table S1. — ICD 10 codes used to define comorbid conditions. (DOCX 19 kb) [file 12891_2016_1354_MOESM1_ESM.docx]

**Table S1. ICD 10 codes used to define comorbid conditions.**

| Comorbid condition | ICD-10 codes (Swedish version) |
| --- | --- |
| Diabetes mellitus | E10-E14, O24 |
| Hypertension | I10-I15 |
| Ischemic heart disease  Unstable angina  Myocardial infarction  Chronic ischemic heart disease  Atrial fibrillation or flutter | I20-I25  I200  I21-I22  I25  I48 |
| Heart failure | I42, I50 |
| Valvular disease | I05-I08, I34-I38 |
| Cerebrovascular disease  Ischemic stroke  Hemorrhagic stroke  Unspecified stroke  TIA | I60-I69, G45  I63  I60-I61  I64  G45 |
| Venous thromboembolic disease  Pulmonary embolus  Deep venous thrombosis | I26, I80-82  I26  I801-I802, I81, I822-I829 |
| Chronic respiratory disease  COPD and chronic bronchitis  COPD and asthma  Interstitial lung disease | J40-J47, J60-J64, J66-J67, J82, J84  J41-J44  J44-46  J841, J849 |
| Chronic renal insufficiency | N18 |
| Depression | F32-F33 |
| Malignancy | C00-C97 |
| Fractures (sites related to osteoporosis) | S220, S320, S422, S525, S720-S721 |
| Infectious disease  Pneumonia  Sepsis | A00-B99, G00-G02, G042, G05-G07, H66-H67, H70, J00-J22, J32, J340, J36, J383, J390-J391, J85-J86, K102, L00-L08, M00-M01, M462-M465, M86, N10, N300, N390  J100, J110, J12-18  A40-A41, A021, A207, A227,A267, A241, A282, A327, A392-A394,A427, A548, B377 |
| Prosthetic surgery  Hip  Knee | **Surgical and treatment codes ^a^**  NFB  NGB |

^a^ Surgical and therapy procedure codes according to a Swedish classification, held by the National board of health and welfare.
